# Loss of *highwire* Protects Against the Deleterious Effects of Traumatic Brain Injury in *Drosophila Melanogaster*

**DOI:** 10.3389/fneur.2020.00401

**Published:** 2020-05-12

**Authors:** Ciaran S. Hill, Jemeen Sreedharan, Andrea Loreto, David K. Menon, Michael P. Coleman

**Affiliations:** ^1^John van Geest Centre for Brain Repair, University of Cambridge, Cambridge, United Kingdom; ^2^The Babraham Institute, Cambridge, United Kingdom; ^3^Institute of Psychiatry, King's College London, London, United Kingdom; ^4^Division of Anaesthesia, Department of Medicine, University of Cambridge, Cambridge, United Kingdom; ^5^Department of Clinical Neurosciences, Wolfson Brain Imaging Centre, University of Cambridge, Cambridge, United Kingdom

**Keywords:** wallerian degeneration, traumatic brain injury, neuroprotection, axons, highwire

## Abstract

Traumatic brain injury is a major global cause of death and disability. Axonal injury is a major underlying mechanism of TBI and could represent a major therapeutic target. We provide evidence that targeting the axonal death pathway known as Wallerian degeneration improves outcome in a *Drosophila Melanogaster* model of high impact trauma. This cell-autonomous neurodegenerative pathway is initiated following axon injury, and in Drosophila, involves activity of the E3 ubiquitin ligase *highwire*. We demonstrate that a loss-of-function mutation in the *highwire* gene rescues deleterious effects of a traumatic injury, including—improved functional outcomes, lifespan, survival of dopaminergic neurons, and retention of synaptic proteins. This data suggests that *highwire* represents a potential therapeutic target in traumatic injury.

## Introduction

Traumatic brain injury (TBI) is defined as an alteration in brain function, or other evidence of brain pathology, caused by an external force (1). It is a major cause of death and disability, and each year in Europe alone ~2.5 million people will experience TBI, 1 million of which will be admitted to hospital, and 75,000 will die (2). Various secondary injury cascades follow the initial primary insult of TBI, promoting ongoing neuronal cell loss. The secondary injury response is multifaceted and comprises a number of processes including Wallerian degeneration (WD) (3).

WD is an active, cell-autonomous death pathway that leads to degeneration of the distal axonal segment following transection (4). In some neuronal subtypes WD following axonal injury may lead to a progressive dying back of the proximal axon segment and death of the neuronal cell body (5). A related process, Wallerian-like degeneration, occurs through the same molecular mechanisms as WD but does not require a complete axonal transection and instead is typically characterized by an impairment of axonal transport (4). WD and Wallerian-like degeneration are both thought to play a role in secondary brain injury following TBI although complete axotomy that occurs at the time of an injury (primary axotomy) is uncommon, and the degree to which sub-axotomy level injuries subsequently impair axonal transport and induce Wallerian-like degeneration remains incompletely characterized (6–10).

Activation of the WD pathway is fundamentally linked to the nicotinamide adenine dinucleotide NAD synthetic pathway (Supplementary Figure 1A). Nicotinamide mononucleotide adenylyl transferase (NMNAT) is a key enzyme in the WD/Wallerian-like degeneration pathway, converting nicotinamide mononucleotide (NMN) to nicotinamide adenine dinucleotide (NAD). NMNAT also displays chaperone function during stress responses (11–15). Following transection, the delivery of the axonal isoform of NMNAT (NMNAT2) from the neuronal cell body to the distal axon is compromised. As NMNAT2 is degraded NMN accumulates and levels of NAD fall (13, 16–18). This results in activation of the toll-like receptor adaptor protein; sterile motif-containing and armadillo-motif containing protein (SARM1) resulting in axon fragmentation (Supplementary Figure 1B) (19–23). This mechanism of axon degeneration is conserved across species. In *Drosophila Melanogaster*, only one isoform of NMNAT is present, this is called dNMNAT (drosophila NMNAT) and serves the functions of mammalian NMNAT 1-3 isoforms, including delay in Wallerian degeneration. NMNAT proteins are evolutionarily conserved and dNMNAT can directly substitute for NMNAT isoforms in mammalian systems (24–28).

Evidence for a role of WD/ Wallerian-like degeneration in TBI has emerged from rodent cortical-contusion injury studies. The first WD gene to be explored in a model of TBI was the slow Wallerian degeneration gene (Wld^s^). This gene encodes a mutant protein (WLD^s^) that can substitute for NMNAT, and Wld^s^ expressing mice demonstrate less motor and cognitive impairment following a cortical-contusion injury (29). Similarly, knockout of SARM1 was associated with reduced neuronal loss and cognitive impairment following CCI (30). These studies suggest that therapeutic modulation of the Wallerian-like degeneration pathway may be possible, and potentially could improve outcomes from TBI (3).

Another tractable context to explore the biology of WD is *Drosophila Melanogaster*. Loss-of-function mutations in the *highwire (hiw)* gene in *D. Melanogaster* are associated with a strong delay in axon degeneration following transection both *in vitro* and *in vivo* (24, 31–33). Delayed WD is also seen with mutations in the mammalian ortholog of *hiw*; *PHR1* (4, 34, 35). The *hiw* gene encodes a large 5233 amino acid protein with E3 ubiquitin ligase activity that modulates levels of dNMNAT (24, 31, 35, 36). *Hiw* also has presynaptic regulatory activity that is required to control excess synaptic growth at the neuromuscular junction (31, 36, 37). To investigate for potential modifiers of brain trauma we utilized a model of high impact trauma (HIT) in *D. Melanogaster* in which WD pathways show extensive conservation with mammalian species (4, 19, 20, 35, 38–40). Given the role of *hiw* in dNMNAT depletion and subsequent axon degeneration, and the assessment that Wallerian-like degeneration may contribute to the secondary brain injury seen in TBI, we hypothesized that flies with a null mutation in *hiw* (*hiw*^Δ*N*^) would show protection against the effects of TBI. We show that *hiw*^Δ*N*^ flies showed relative protection against long-term mortality and cell death. Brain vacuolation and necrotic cell death occurred regardless of genotype—suggesting that the *hiw* mutants still underwent a neurodegenerative process but there was reduced presynaptic marker depletion and dopaminergic (PPL1) neuron loss. This suggests that a subset of vulnerable—but functionally important- neurons may be rescued by *hiw* loss-of-function. This translated to a preservation of normal behavioral measures. These findings suggest that *hiw* and its mammalian ortholog PHR1 are potential therapeutic targets in experimental and clinical TBI.

## Materials and Methods

### Drosophila Melanogaster Stocks and Conditions

*Hiw* mutant *hiw*^Δ*N*^ and control *hiw*^*WT*^ (FRT^19A^) flies were obtained from Marc Freeman (University of Massachusetts). Newly enclosed flies were collected daily, separated by sex, into vials of 20–35 flies, and aged for experimental use. All experiments were conducted on flies aged 1–4 days unless otherwise stated. All flies were maintained at a constant 25°C temperature and humidity, in glass vials with standard agar/cornmeal/yeast feed. Flies were exposed to a 12 h light-dark cycle. Feed was changed in all vials once every 14 days or sooner as required. All experiments were conducted exclusively on male flies in order to avoid confounding effects relating to the female reproductive cycle.

### High Impact Trauma Device, Injury Calibration, Incapacitation Rates, and Intestinal Barrier Dysfunction

Flies were subjected to a standardized impact with the HIT device. After injury, vials were laid on their side and flies were given a minimum of 10 min to recover motility before being transferred to a glass vial containing standardized feed. All polystyrene vials were discarded after a single use. The severity of injury was calibrated in *hiw*^*WT*^ flies by assessing the death rates 24 h following HIT when the angle of initial deflection, and thus recoil force, was adjusted. Incapacitation rates were recorded by assessing the percentage of flies that failed to show signs of purposeful movement within 20 seconds of initial impact. To evaluate intestinal barrier dysfunction following HIT flies were transferred to feed containing dissolved Brilliant Blue FCF dye (#80717, Sigma). After 24 h the percentage of flies that had blue food dye dispersed outside of the abdominal cavity and proboscis were counted.

### Early Death Rate and Long-Term Survival Assay

To assess for variation in early death rates we exposed flies to a HIT at a standardized time of day (09:00 h). Any flies dying immediately or within the first 24 h of a HIT were considered to have died of the undifferentiated primary effects of a HIT. Dead flies were removed and all remaining live flies were transferred to new vials and long-term survival was monitored. A daily count of number of fly deaths was conducted in all vials for the lifetime of all flies. Dead flies were discarded every day.

### Rapid Iterative Negative Geotaxis (RING) Assay and Flight Assay

A custom made rapid iterative negative geotaxis (RING) device was manufactured and used to measure negative geotaxis/climbing ability as a behavioral measure of motor function (41, 42). Flies were gently transferred to fresh empty polystyrene vials without anesthesia with a maximum density of 25 flies per vial. Groups of up to 6 vials were inserted into the RING device, and after 5 min for the flies to adjust to the environmental change the device was tapped three times to settle flies to the bottom of the vials. Exactly 5 s after the last tap a photograph was taken to assess the height climbed. The head of the fly was the reference point for the climbing height achieved. Maximum height achieved was graded into 5 mm intervals, flies that climbed <5 mm were scored zero, and any fly that exceeded 50 mm was awarded the maximum score was 5 cm. The average height achieved for the vial was calculated. This was repeated 3 times at 60 s intervals and an average score given for that vial. The climbing ability was calculated on a vial by vial basis. For the flight assay, flies were anesthetized on ice for exactly 5 min then the flat of a 30G 1” needle (#Z192368, Sigma) was attached to the anterior notum of a fly just posterior to the neck using clear nail varnish, leaving flight muscles unimpeded. Flies were given 15 min to fully recover. Needles were fixed in place under a video microscope. If required then a gentle mouth-blown puff of air was used to stimulate flight and the flying time was recorded for 30 s per fly for analysis. This was repeated 3 times per fly and the average of time spent in flight was calculated for each condition. This was a terminal assay. We maintained standardized conditions using the same experimental setup for each range of experimental condition in both the climbing and flying assay. The same experimenter conducted all experiments in a standardized fashion, in a single laboratory room, at the same time of day (09:00), and with the same equipment and environmental conditions—including room lighting and 25 degrees temperature).

### Haematoxylin and Eosin Histology, and Vacuole Counting

Anesthetized flies were submerged in cold 1x PBS, the proboscis and rostral trachea were removed, and the amputated heads gently rocked in fresh ice cold 4% paraformaldehyde solution for 45 min. Brain extraction is not necessary for high-quality H&E analysis so brains were stained *in situ* within the cuticle. The tissue was alcohol dehydrated, xylene washed, and embedded in paraffin for serial sectioning with a microtome (Leica RM2235) at a thickness 7 μM. Sections were mounted on poly-L-lysine coated slides (P0245, Sigma). Wax was removed with a xylene bath then alcohol washes before haematoxylin and eosin staining, and application of coverslips. After blinding, three representative coronal sections were examined from a central brain region that included the medulla using brightfield microscopy. The average number of ≥5 μM vacuoles per slice in each brain was calculated. Vacuoles were measured at the greatest diameter—microscopy photographs were analyzed using image J software (v1.51n). All image analysis was performed with the investigator blinded to experimental conditions.

### Immunohistochemistry

Fly brains were dissected in cold 1x PBS and fixed in 4% paraformaldehyde-PBS for 30 min. Brains were dissected for immunohistochemical analysis as penetration of antibody through the waxy cuticle is limited and would otherwise be inaccurate. Samples were washed in 1x PBS with 0.3% Triton X-100 (#T8787, Sigma) and blocked for 1 h at room temperature in 1x PBS with 0.3% Trition X-100 and 1% BSA (#9647, Sigma). Brains were incubated in primary antibody diluted with blocking solution for 72 h. After washing and incubating in a fluorescent secondary antibody solution for 4 h, samples were washed and mounted between two coverslips in ProLong diamond antifade mountant (#P36965, ThermoFisher). Confocal images were acquired on a Leica imaging system, at z-stack intervals not greater than 0.6 μM and blinded for analysis. Primary antibodies used were mouse Tyrosine-Hydroxylase (anti-TH) antibody 1:100 (TH-antibody, #22941, Immunostar Inc.) for the PPL1 cluster. Secondary antibodies were goat anti-mouse IgG (H+L) Alexa Fluor 488 (#A11034, ThermoFisher), and donkey anti-rabbit IgG (H+L) Alexa Fluor 594 (##21207). All image analysis was performed with the investigator blinded to experimental conditions. For each brain a Z-stack was taken that covered the entire extent of the fixed brain. Each Z-stack was set at a slice thickness of 0.5 μm. There were 1 or 2 regions of interest per brain depending on the quality of dissection. The PPL1 cluster is identified by its location and its characteristics major neuropil projections to the mushroom bodies and vicinity, this is clearly different from neurons in other adjacent clusters (43). A total of 10–12 PPL1 clusters were used per condition, this equates to 5–12 brains. All brains were immunolabelled using a standardized method in parallel at a single time point. Image settings were kept constant throughout all analysis. Images were analyzed using image J software (v1.51n).

### Immunoblotting

Fifteen whole fly heads were collected and homogenized in Laemmli sample buffer and centrifuged 13,000 rpm for 5 min. Supernatant protein lysates were resolved by SDS-PAGE on Mini-protean 4-15% SDS resolving gel (#4561086, Bio-Rad) and transferred to Immobilon-P PVDF membrane (#IPVH00010, Merck). They were blocked in a 1% BSA solution (#9647, Sigma), then probed with the following primary antibodies: Bruchpilot 1:5000 (nc82, #2314866, DSHB), Discs-large 1:10,000 (4F3, #528203, DSHB), Neuroglian 1:5000 (BP104, #528402, DSHB), and β tubulin 1:5000 (E7, #2315513, DSHB). Bands were detected with goat anti-mouse, and goat anti-rabbit horseradish peroxidase-linked secondary antibodies (#1706515 and #1721011, Bio-Rad) and Supersignal West Dura extended duration chemiluminescence substrate (#34075, ThermoFisher). Immunoblotting was done in triplicate. Density of bands was quantified using Image J software (v1.51n). Analysis method included cropping a standard size rectangular band around the area of the blot of interest was selected and then the generated histogram was cropped with a straight line at the base of the intensity peak—the resulting area under the curve was then converted by image J to a numerical value which represented blot density and was used for data analysis. Blot density was compared to the density of the β tubulin loading control.

### Flow Cytometry

Five fly brains were dissected in cold 1xPBS solution and transferred to dissection media containing 7.5 mls of DMEM (high glucose, HEPES, phenol-red free, #21063029, ThermoFisher), 2.5 mls of 10x trypsin (#15400054, ThermoFisher), and 1% BSA (#9647, Sigma). Brains were washed in trypsin free dissection media, gently triturated using a 200 μL pipette 30 times, then filtered through a 70 μM strainer. The resulting solution was mixed in a 1:1 ratio with Annexin V & dead cell solution (Annexin V & 7-AAD, #MCH100105, Merck), incubated at room temperature for 20 min, then processed on the Muse Cell Analyser (Merck) using inbuilt analysis software. Dilutions in trypsin free dissection media we made as required. For positive controls brains were incubated in 200 mM of Actinomycin D (#A1410, Sigma) at 37°C for 6 h before processing. The average cell/ml concentration was 3.0 x 10^5^ with a range of 2.1 x 10^5^ to 9.8 x 10^5^ between groups. The Muse™ Cell Analyzer system can detect cells sized between 2 and 60 μm. The flow cytometry data was obtained from 5,000 events (gated cells) per sample. The percentages of cells shown in the figures were calculated from the mean fluorescence intensity in each of the four quadrants. Cell size was thresholded at a cell size index of 1.5 to exclude cellular debris. Gating was kept constant for all experiments—it was set on the two-parameter dot plot as displayed. This is auto-optimized by the Muse™ Annexin V & Dead Cell Kit (EMD Millipore).

### Statistical Analysis

Statistical analysis and graph fitting was performed using Graphpad Prism 7.02 (GraphPad Software Inc.). Statistical tests and biological/technical repeats are listed in respective figure legends—as a minimum all experiments were repeated in triplicate. Significance threshold was taken as a *p* value < 0.05.

## Results

### A *Drosophila melanogaster* High Impact Trauma Device Produces a Quantifiable Injury That Demonstrates Genotype-Dependent Variation in Early Survival

To model TBI in *D. Melanogaster* we utilized the HIT device. This consists of a spring-loaded attachment that holds a polystyrene vial of flies and inflicts a rapid acceleration-deceleration impact injury [Supplementary Figure 2A—image from Katzenberger et al. (38)]. The severity of injury correlated with the angle of initial deflection of the device spring. An angle of 90° produced an average death rate of 22% at 24 h and 30% at 7 days post-injury in wild type (*hiw*^*WT*^) flies (Supplementary Figure 2B). External injuries were never seen at angles of 90° or less but were common (82%) at an angle of 135°, therefore the 90° was chosen to represent a single severe HIT in subsequent studies. Our choice of HIT severity was also based on the observed incapacitation rate–defined as the proportion of flies that demonstrated a lack of purposeful movement 20 s or more following trauma. The incapacitation rate was comparable in both *hiw*^*WT*^ and *hiw*^Δ*N*^, but when compared to a HIT at 90^o^, incapacitation rate at 70^o^ was greatly reduced (~100% vs. ~50%) and was less consistent (Supplementary Figure 2C). Given this more consistent evidence of neural injury, and the lack of evidence of systemic injury at 90^o^, this HIT protocol was therefore chosen to represent a single severe traumatic HIT in subsequent studies. In order to minimize variability a single experimenter used the same HIT device with the same setup for all impact experiments. A visual marker was placed at the 90^o^ position (when viewed from a lateral viewpoint) this allowed an exact reproducible spring angle each time.

The *hiw*^Δ*N*^ flies have previously been reported as having synaptic overgrowth at neuromuscular junctions but are otherwise phenotypically normal (31, 36, 37, 44). We found that in uninjured flies the genotype (*hiw*^*WT*^ or *hiw*^Δ*N*^) did not have any influence on long-term survival, indicating no significant effect of the *hiw* null allele on baseline viability. HIT resulted in an incapacitation rate that was the same in both *hiw*^*WT*^ and *hiw*^Δ*N*^ and 24 h following a HIT both *hiw*^*WT*^ and *hiw*^Δ*N*^ flies demonstrated reduced survival compared to uninjured flies (Supplementary Figure 2D). However, notably the early death rate was greater in *hiw*^Δ*N*^ flies. The cause of this small excess burden of death was not determined by this study. This may reflect non-TBI related causes related to the constitutive loss of *hiw* expression. Increased mortality following HIT could also be due to traumatic intestinal barrier dysfunction. To exclude this possibility flies were exposed to colored food post HIT and examined for leakage of food dye (45). This demonstrated very low rates of intestinal barrier breakdown regardless of genetic background, supporting the conclusion that the HIT severity we applied did not cause gross thoraco-abdominal trauma (Supplementary Figure 2E).

### High Impact Trauma Induced Long-Term Mortality Is Reduced and Functional Impairment Rescued by Loss of *highwire*

**Figure 1:**
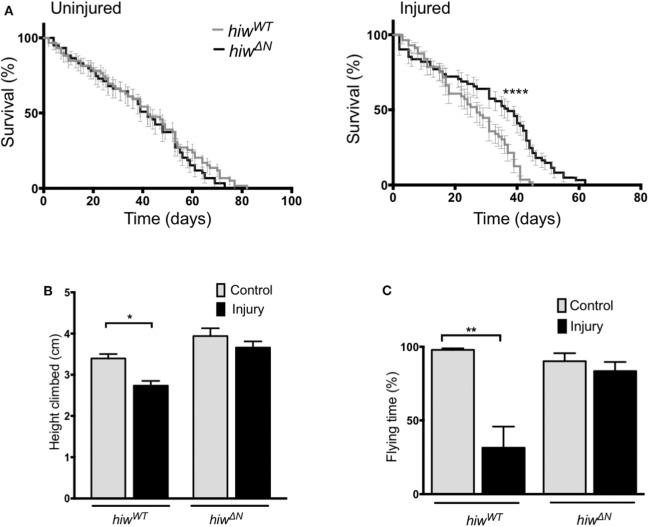
Long-term mortality, climbing, and flying ability are relatively preserved in *highwire* mutants. **(A)** Mortality rates over lifetime of flies in hiw^Δ*N*^ and hiw^WT^ flies. *n* = 6 vials of 20-35 flies per condition. Statistical analysis was with Log-rank test. ****p ≤ 0.0001. **(B)** Reduction in climbing ability compared to baseline in injured versus uninjured hiw^Δ*N*^ and hiw^WT^ flies at 45 days post injury. *n* = 6 vials of 35 flies per condition. Statistical analysis was with two-way ANOVA test. *p ≤ 0.05. **(C)** Percentage of time spent in flight over a 30 s period in injured versus uninjured hiw^Δ*N*^ and hiw^WT^ flies at 7 days post injury. *n* = 10-12 per condition. Statistical analysis was with two-way ANOVA test. **p ≤ 0.01. Error bars show standard error of the mean for all experiments.

To determine if loss of *hiw* could protect against long-term effects of TBI we examined survival in animals that lived beyond the initial 24 h post injury period. Injury caused a significant reduction in long-term survival in both *hiw*^*WT*^ and *hiw*^Δ*N*^ animals compared to uninjured controls. However, *hiw*^Δ*N*^ animals demonstrated significantly increased survival following injury compared to *hiw*^*WT*^, particularly from ~20 days post injury (Figure 1A). To determine if loss of *hiw* could rescue injury-induced climbing deficits we used the rapid iterative negative geotaxis (RING) assay (Supplementary Figure 2F) (41, 42). This demonstrated a significantly reduced climbing ability after HIT in *hiw*^*WT*^ flies, which was attenuated in those with a *hiw*^Δ*N*^ deletion (Figure 1B). We also examined motor function by measuring flying ability. Flight behavior has been shown to vary with the level of protocerebral anterior medial dopaminergic neurons (46). At 7 days following injury *hiw*^*WT*^ show a significant reduction in the percentage of time they spend in flight over a 30 s period when compared to controls of their own genotype. This deterioration in flying activity after injury is strikingly diminished in the *hiw*^Δ*N*^ flies (Figure 1C). Injured flies that do not have flight activity still have healthy looking wings, make frequent spontaneous wing movements, and engage in wing grooming behavior, suggesting that the failure to fly even when provoked by a stimulus of air is not simply as a result of a peripheral wing injury. This is supported by the cases where short periods of flight are initiated but the flies seem unable to maintain for a prolonged duration.

### Loss of *highwire* Reduces Neuronal Apoptosis

**Figure 2:**
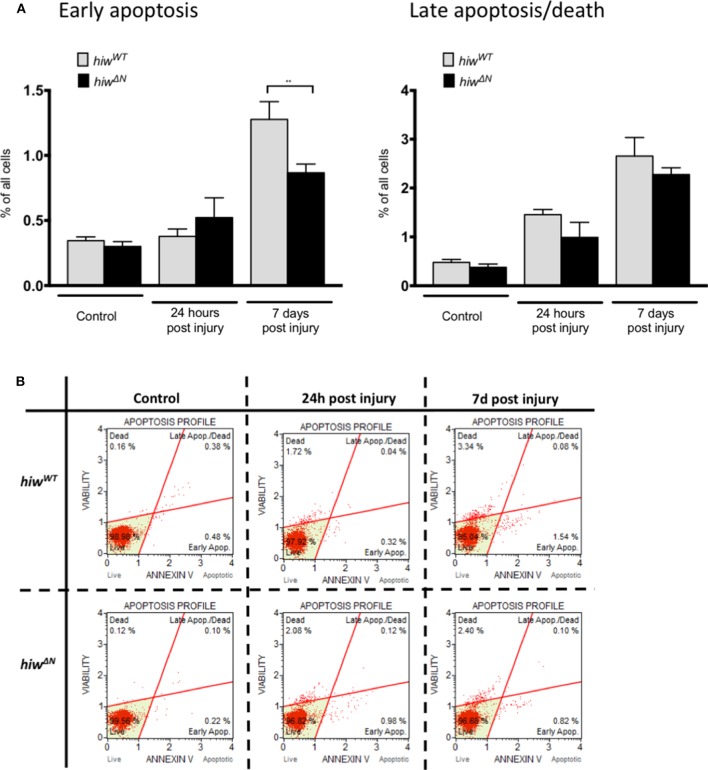
Flow cytometry of dissociated drosophila brains shows necrosis but low levels of apoptosis after injury. **(A)** Flow cytometry demonstrating the percentage of cells undergoing early apoptosis, and late apoptosis/cell death following injury at 24 h and 7 days in hiw^Δ*N*^ and hiw^WT^ flies. *n* =9. Error bars show standard error of the mean. Statistical analysis was with two-way ANOVA test. **p ≤ 0.01. **(B)** Graphical representative flow cytometry data.

In order to explore the mechanism of neuronal cell loss following HIT we performed flow cytometry of dissociated fly brains (Figures 2A,B). This demonstrated low-levels of baseline early apoptosis (Annexin V positive, 7 AAD negative) and late apoptosis/cell death (Annexin V positive and 7 AAD positive) in *hiw*^*WT*^ and *hiw*^Δ*N*^ uninjured controls (<0.5%). In contrast, 7 days following injury there was a rise in both early apoptosis, and late apoptosis/cell death by flow cytometry. This is direct evidence for brain injury following impact with the HIT device. The levels of early apoptosis were significantly lower in the *hiw*^Δ*N*^ flies compared to *hiw*^*WT*^ following injury at 7 days, however the absolute difference was small (~0.5%). There were no significant differences in late apoptosis/death at either time point. This suggests that HIT causes a neuronal apoptotic cell death that is rescued by *hiw*^Δ*N*^ mutation.

### Dopaminergic Neuron Loss Is Attenuated by Loss of *highwire*

**Figure 3:**
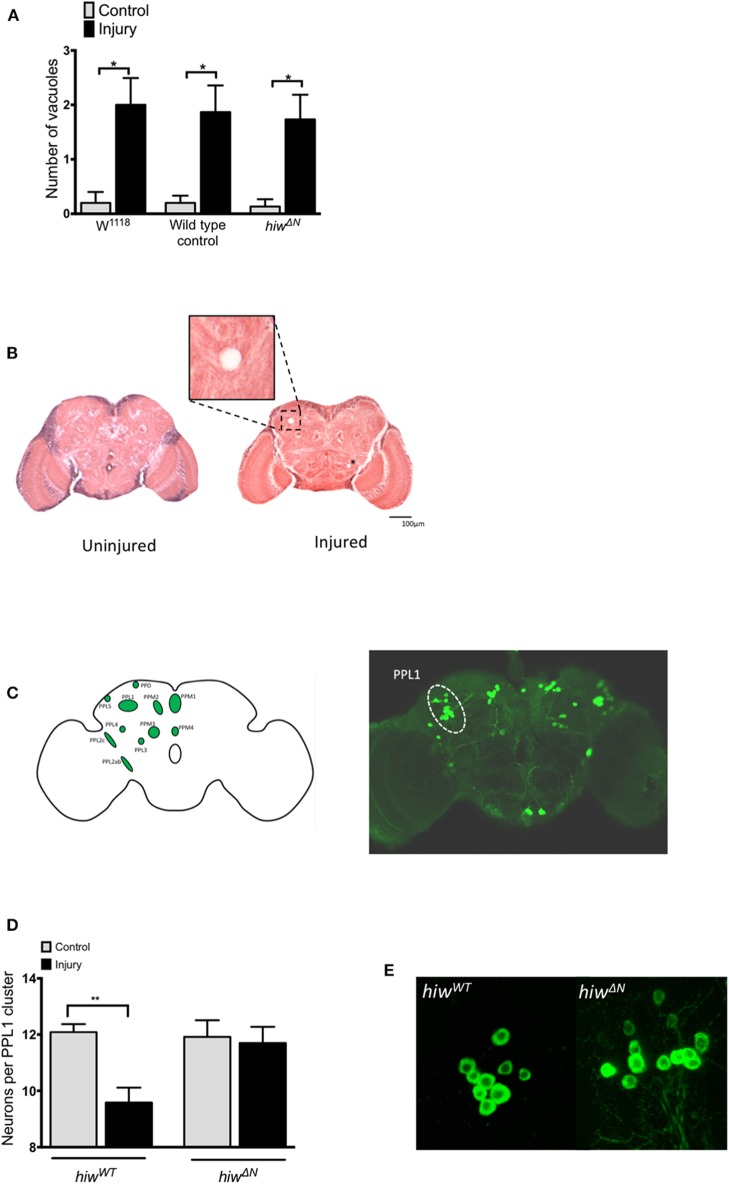
Injured flies develop brain vacuoles regardless of genotype, but depletion of PPL1 cluster dopaminergic neurons that is prevented by *highwire* mutation. **(A)** Rates of vacuolation seen by hematoxylin and eosin staining in central brain regions at 28 in hiw^Δ*N*^ and hiw^WT^ flies 28 days following injury. *n* = 5 per condition. Error bars show standard error of the mean. Statistical analysis was with two-way ANOVA test. *p ≤ 0.05. **(B)** Representative hemotoxylin and eosin stained brain sections from uninjured and injured hiw^WT^ flies. The insert shows a close up of a typical vacuole. A further small vacuole is marked by an asterix. **(C)** Schematic image and TH stained whole brain mount showing the location of various dopaminergic neuronal groups including the PPL1 cluster. **(D)** Number of TH-positive PPL1 neurons in hiw^Δ*N*^ and hiw^WT^ flies at 28 days post injury. *n* = 10-12 clusters per condition. Error bars show standard error of the mean. Statistical analysis was with two-way ANOVA test. **p ≤ 0.01. **(E)** Representative PPL1 dopaminergic neuronal clusters showing depleted neuron numbers in injured hiw^WT^ flies (9 neurons) and preserved numbers in injured hiw^Δ*N*^ flies (12 neurons).

To explore the mechanisms underlying the protective effects of *hiw* following TBI we performed histological studies on fly brains. We first looked for evidence of injury-induced neurodegeneration by quantifying brain vacuolation (38, 47–49). Both *hiw*^*WT*^ and *hiw*^Δ*N*^ flies demonstrated an increase in vacuolation at 28 days following injury, though no attenuation of vacuolation was observed in *hiw*^Δ*N*^ flies (Figure 3A). We next examined a well-characterized subpopulation of dopaminergic neurons (protocerebral posterior lateral 1 cluster, PPL1), which are involved in climbing and flying behavior (Figure 3B) (46, 50, 51).

The number of neurons in the PPL1 cluster is remarkably consistent in wild-type flies, with an average of 12 neurons. Immunostaining for tyrosine hydroxylase (TH) demonstrated a significant decrease in the number of neurons in the PPL1 cluster following injury in *hiw*^*WT*^ flies. However, no reduction in PPL1 neuron numbers was seen following injury in *hiw*^Δ*N*^ flies (Figures 3C,D).

### Loss of *highwire* Reduces Synaptic Protein Loss Following Injury

**Figure 4:**
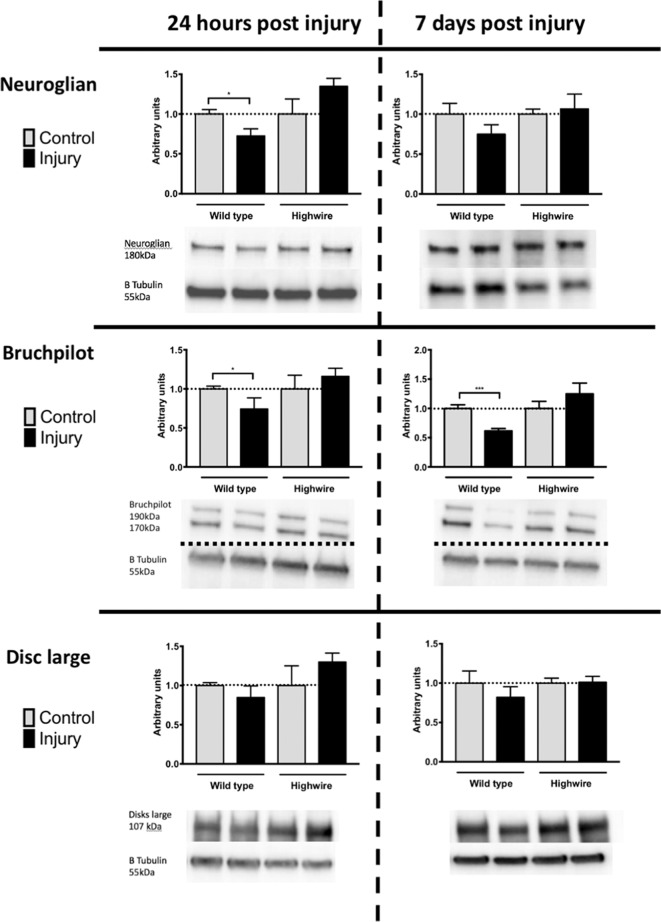
Injured flies demonstrate a loss of presynaptic marker protein that is prevented by *highwire* mutation. Levels of Neuroglian, Bruchpilot, or Disc large relative to β tubulin loading control at 24 h and 7 days following injury in hiw^Δ*N*^ and hiw^WT^ flies. Representative blots are displayed. *n* = 5 per condition. Error bars show standard error of the mean. Statistical analysis was with two-way ANOVA test. *p ≤ 0.05, ***p ≤ 0.001.

In order to probe the nature of underlying cellular responses to the HIT we conducted western blots analysis using the pan-neuronal marker Neuroglian (Ngl), the presynaptic marker Bruchpilot (Brp), and the post synaptic maker Disc Large (Dlg) at 24 h and 7 days following injury. After injury there was a significant reduction in Ngl at 24 h in *hiw*^*WT*^ indicating a generalized loss of neurons. This trend was also seen at 7 days but was no longer significant. In contrast, *hiw*^Δ*N*^ flies did not have a reduction in Ngl. Mirroring the Ngl loss, Brp was also reduced following injury at 24 h, but this reduction persisted at 7 days with some progressive loss. As with ngl, the *hiw*^Δ*N*^ flies were protected against Brp loss regardless of timepoint- and a non-significant trend suggesting a possibly increase was seen. Finally, Dlg levels were examined. Again, trends mirrored those of Ngl and Brp, with small falls seen in the *hiw*^*WT*^, and small rises in the *hiw*^Δ*N*^ flies, but notably these were not significant in any case (Figure 4).

## Discussion

Our results provide support for the hypothesis that a mutation in the *hiw* gene (*hiw*^Δ*N*^) demonstrates protection against the deleterious effects of HIT. The *hiw*^Δ*N*^ mutation results in a complete loss-of-function of *highwire*, leading to delayed degradation of dNMNAT, a core step in the WD process. The *hiw*^Δ*N*^ mutation has previously been shown to strongly delay WD both *in vitro* and *in vivo* (24, 31–33). In *D. Melanogaster*, the *hiw*^Δ*N*^ mutation protected against several deleterious effects of a high-velocity impact trauma model. Deaths were significantly reduced in keeping with longer-term protection against secondary brain injury processes. Flies with *hiw*^Δ*N*^ mutation suffer less neurodegeneration following HIT as manifest by reduced PPL1 dopaminergic neuron loss. Dopaminergic neurons have previously been shown to demonstrate selective vulnerability following brain injury and in various neurodegenerative conditions including Parkinson's disease (50–53). Our investigation of dopaminergic neurons was limited to the PPL1 subpopulation. Although the TH stain marks other dopaminergic populations the PPL1 population is the best characterized and has the small variability in its neuronal number. This is not the case for other neuronal populations—which are less consistent in their number, not as easy to reliably count due to variability in density, and not as well characterized in terms of relationship to function. The focus on PPL1 allowed us the confidence that our staining technique was reliable and comparable to pre-existing studies, and allowed us to robustly detect differences due to injury or genetic modification. However, as a result of this specific focus we cannot make comment about the effect on other neuronal populations. In order to try and address the limitation of only quantifying a small subpopulation we looked for more general evidence of neuronal loss and rescue with the vacuolation assay. Injury resulted in increased vacuolation in all genotypes but interestingly this was not rescued in the *hiw*^Δ*N*^ mutants.

Given the well-characterized function of *highwire* in dNMNAT degradation, the mechanisms for the protection seen with *hiw*^Δ*N*^ mutation are likely to directly involve the Wallerian-like degeneration pathway, specifically through maintenance of dNMNAT levels in the axonal compartment and/or the cell body and delayed neuronal degeneration. As there is only a single dNMNAT isoform in *D. Melanogaster*, a decline in levels may cause death of both the axon and the cell body. An important future experiment would be to assess dNMNAT levels in a neuronally expressed HA-nmnat tagged fly system.

An alternative explanation would be that loss of *highwire* function is maintaining dNMNAT levels that are then functioning as a molecular chaperone, possibly through alleviation of prototoxic stress (11, 12, 14, 15, 54). Given the nature of the model system we cannot completely exclude that the rescue of lifespan and behavior are not due to effects outside of the CNS, however, the lack of external injuries, the failure of the model to cause intestinal barrier dysfunction, and the initial period of recovery and normal behavior following injury argue against this. The *hiw*^Δ*N*^ mutation is constitutive, therefore there may be unrecognized systemic effects beyond those we have characterized in the brain. This could be addressed in future studies by examination of a conditional mutant. We know that *hiw*^Δ*N*^ flies have previously been reported as having synaptic overgrowth at neuromuscular junctions but are otherwise phenotypically normal (31, 36, 37, 44). We cannot completely exclude the possibility that there is a hitherto unrecognized underlying phenotypic difference that we, and previous researchers have failed to identify. As long as this possibility remains we would urge caution in interpreting the results of this study in isolation.

We did not find any alteration in the levels of synaptic proteins in the uninjured *hiw*^Δ*N*^ brains compared to wild-types in our analysis. This suggests that the synaptic overgrowth effects seen previously at the neuromuscular junction of *hiw*^Δ*N*^ do not occur in the brain. We demonstrated that HIT trauma resulted in an excess of apoptotic cell death that was partially rescued by *hiw*^Δ*N*^. This is an intriguing finding given that WD is a non-apoptotic mechanism of axon degeneration. One possibility is that absence of retrograde delivery of trophic factors by axonal transport could trigger apoptosis of cell bodies—this hypothesis requires further testing. Behavioral measures are less commonly used and well-defined in flies compared to mammalian systems. We chose to use two of the commonest and best characterized in climbing and flying assays. The ability of *hiw*^Δ*N*^ to prevent injury induced climbing and flying defects may be explained by dopaminergic neuron rescue, and PPL1 neuronal loss has previously linked to climbing deficits (46, 51). The preferential presynaptic protein loss seen on Western blot analysis is a pattern that is established in neurodegenerative models (55, 56). dNMNAT has also been shown to maintain presynaptic active zones by directly interacting with bruchpilot (57). This is consistent with our finding that bruchpilot was relatively preserved after injury in *hiw*^Δ*N*^. Together, the finding suggest that HIT causes apoptotic and selective neuronal cell death and presynaptic protein loss, and that *highwire* loss of function can partially rescue these effects and their functional consequences. This helps establish *highwire* as a potential therapeutic target in brain trauma.

## Data Availability Statement

All datasets generated for this study are included in the article/supplementary material.

## Ethics Statement

The experimental protocol was approved by the Babraham Institute ethical review board, and all animal work was performed in accordance with the 1986 Animals (Scientific Procedures) Act under Project License PPL 70/7620 following an appropriate ethical review process at the Babraham Institute. No live vertebrates or higher invertebrates were used in this research.

## Author Contributions

CH, JS, AL, DM, and MC conceived the study, designed the experiments, and wrote the manuscript. CH performed all experiments.

## Conflict of Interest

The authors declare that the research was conducted in the absence of any commercial or financial relationships that could be construed as a potential conflict of interest.
