# Supplementary figures and images for: Loss of highwire Protects Against the Deleterious Effects of Traumatic Brain Injury in Drosophila Melanogaster
Source: Front Neurol. 2020 May 12;11:401. doi: 10.3389/fneur.2020.00401 (PMC7235382; doi:10.3389/fneur.2020.00401)

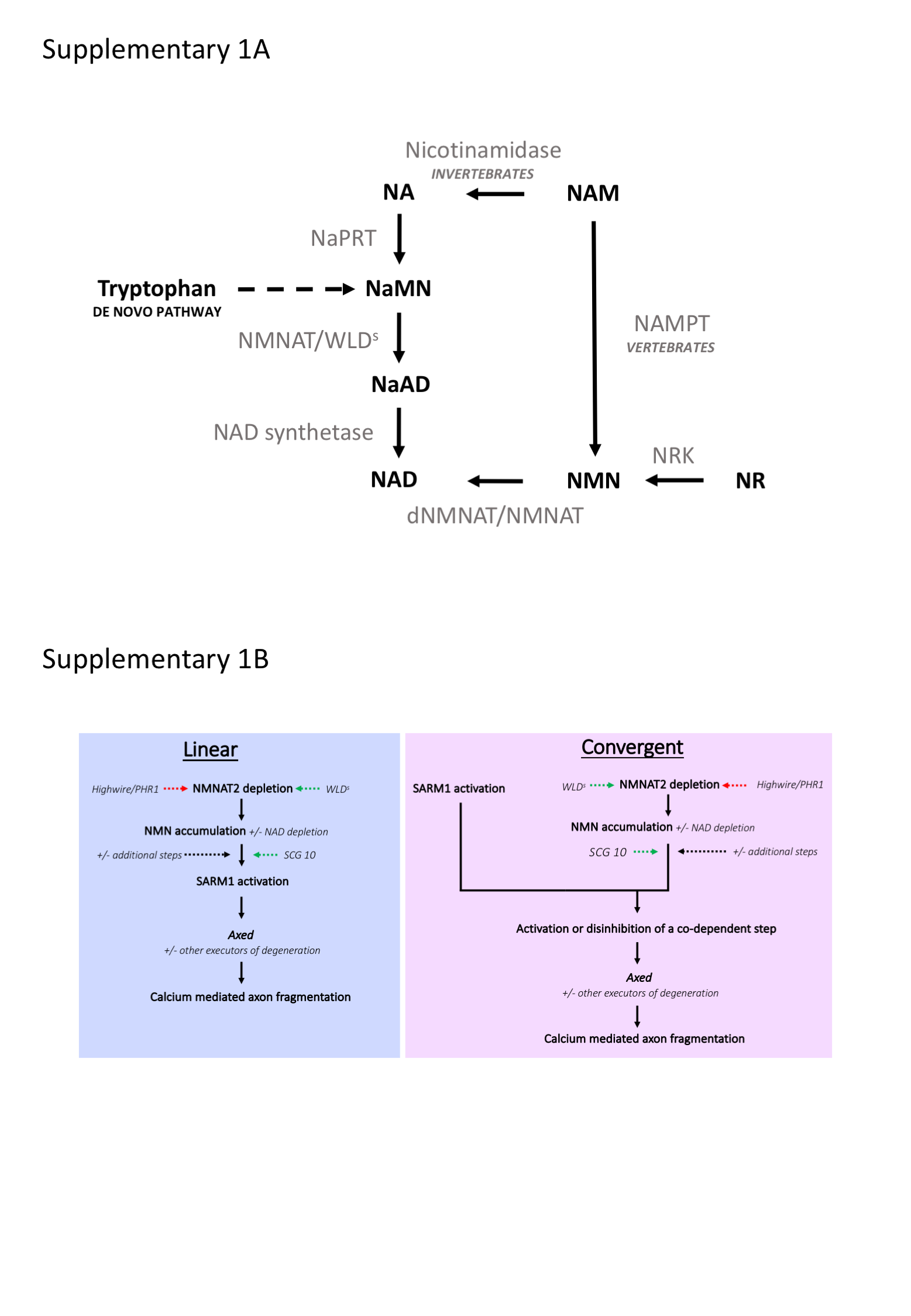

Supplement: Supplementary file 1 [file Image_1.TIFF]

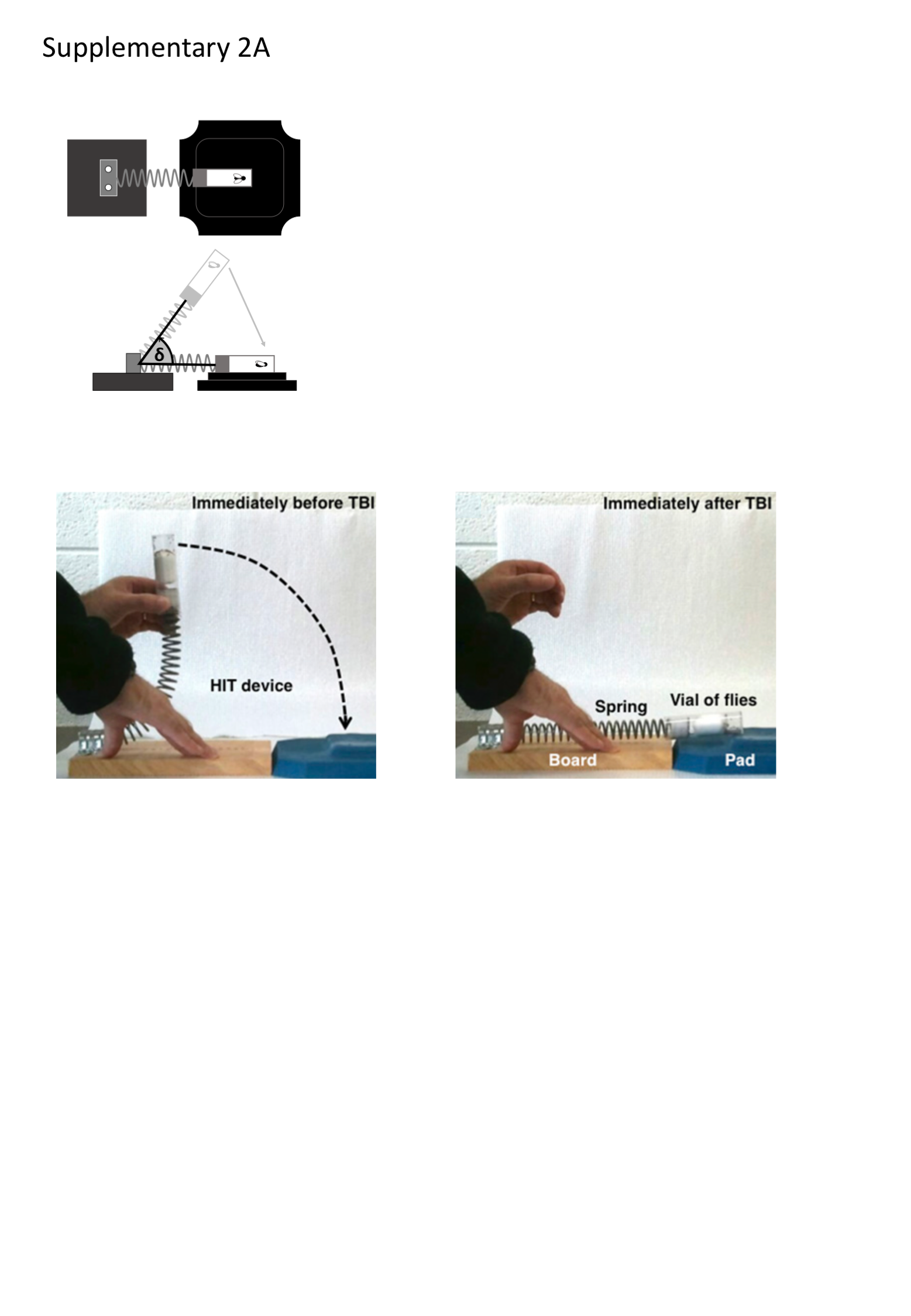

Supplement: Supplementary file 2 [file Image_2.TIFF]

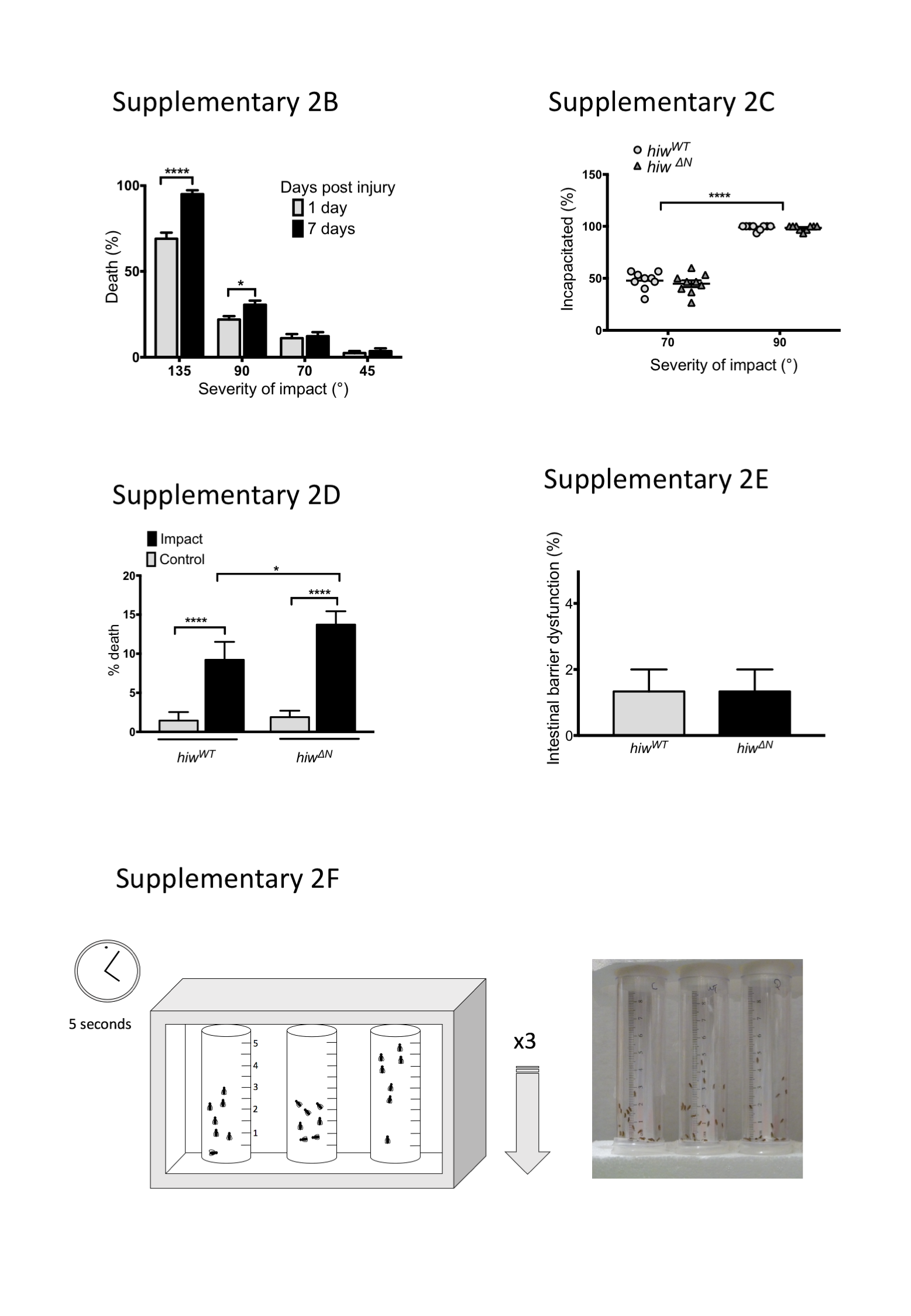

Supplement: Supplementary file 3 [file Image_3.TIFF]
