# Supplementary material for: Loss of highwire Protects Against the Deleterious Effects of Traumatic Brain Injury in Drosophila Melanogaster
Source: Front Neurol. 2020 May 12;11:401. doi: 10.3389/fneur.2020.00401 (PMC7235382; doi:10.3389/fneur.2020.00401)
Supplement: Supplementary file 4 [file Table_2.DOCX]

**Supplementary Figure 1A**

**The NAD synthesis pathway**

The de novo NAD synthesis pathway generates NAD from the essential aromatic amino acid tryptophan. Nicotinic acid mononucleotide (NaMN) is converted to nicotinic acid adenine dinucleotide (NaAD) by nicotinamide mononucleotide adenylyltransferase (NMNAT). NaAD is then converted to NAD by NAD synthetase. There are three additional thee salvage pathways. Nicotinamide mononucleotide (NMN) is produced by nicotinamide phosphoribosyltransferase (NAMPT) from nicotinamide (NAM), and by nicotinamide riboside kinase (NRK) from nicotinamide riboside (NR). NMN is then converted to NAD by NMNAT. Alternatively, NAM is converted to nicotinic acid (NA) by nicotinamidase, and then NA is converted to NaMN by nicotinc acid phosphoribosyltransferase (NaPRT).

**Supplementary Figure 1B**

**Molecular control pathways of Wallerian degeneration**

Experimental evidence has thus far not been able to definitively differentiate between the possibilities of a linear or a convergent Wallerian degeneration pathway. Steps in WD include NMNAT2 depletion, NMN accumulation, prodegenerative SARM1 activation with subsequent Axed activation (in Drosophila), and finally calcium mediated axon dismantling. SARM1 activation may be a linear downstream consequence of NMN accumulation/a changing ratio of NMN/NAD, or SARM1 may function in parallel with a NMNAT2 depletion dependent process to converge on a co-dependent step. **Black arrows** signify the core pathway, **red arrows** signify pro-degenerative modulating factors, while **green arrows** represent factors that delay Wallerian degeneration. It is likely that this picture is incomplete and additional steps will be elucidated in the future. Adapted from Conforti et al & Gilley et al.^12,18^

**Supplementary Figure 2**

**Rates of death following injury relate to the severity of a single impact as determined by the initial angle of spring deformation and by genotype, incapacitation rates vary with force but not genotype, and the intestinal barrier is rarely disrupted following a single impact**

**(A)** A top-down and lateral schematic of the high-impact trauma (HIT) device (left) and photographic image showing device in use (right)Varying the angle of initial deflection (δ) alters the amount of stored energy and hence the restoring force upon release of the spring. When the released vial strikes the impact board the flies are subjected to a rapid deceleration force proportional to δ. **(B)** The rapid iterative negative geotaxis (RING) device consists of a custom made polystyrene container that allows insertion of clear polystyrene vials of flies. These are firmly held in place between the polystyrene floor and ceiling of the box, this close fit also prevents escape of flies from the vials. Directly behind each vial is a measuring scale that marks out centimeters in 1mm intervals, this is visible through the vials and allow direct measurement of a fly’s height at any given timepoint. A schematic and photograph of the RING is presented. **(C)** Percentage survival one day and seven days post injury was assessed in relation to the severity of a single impact. Severity was altered by adjusting the degree of initial spring deformation calculated from the horizontal in the HIT. n = 9 vials of 20-35 flies per condition. Error bars show standard error of the mean. Statistical analysis was with two-way ANOVA test. *= p≤0.05, **** = p≤0.0001. **(D)** All genotypes tested showed a significant increase in mortality 24 hours following a single severe TBI compared to matched controls. 24 hour mortality was greatest in the injured hiw^ΔN^ flies, which were significantly more likely to die than injured hiw^WT^ flies. n = 6 vials of 20-35 flies per condition. Error bars show standard error of the mean. Statistical analysis was with two-way ANOVA test. *= p≤0.05, **** = p≤0.0001. **(E)** Incapacitation rates were determined by subjecting flies to a single impact and assessing the percentage making no purposeful movement 20 seconds later. Each symbol represents the average incapacitation rate for one vial of flies following an impact at either 70° or 90° (70° v 90°**** p ≤0.0001) n = 9 vials of 20-35 flies per condition. Error bars show standard error of the mean. Statistical analysis was with two-way ANOVA test. **(F)** Following a single impact flies were fed standard feed containing blue food dye. In the event of intestinal barrier dysfunction the dye would be expected to extend beyond the gut and proboscis, manifesting as a diffusely blue fly.  n = 9 vials of 20-35 flies per condition. Error bars show standard error of the mean. Statistical analysis was with Mann-Whitney U test.
